# Annual Meeting of the Association of Medical Officers of Asylums and Hospitals for the Insane

**Published:** 1857-10-01

**Authors:** 


### Art. X.—ANNUAL MEETING OF THE ASSOCIATION OF MEDICAL OFFICERS OF ASYLUMS AND HOSPITALS FOR THE INSANE.

The annual meeting for 1857 was held in London, on the 2nd day of July, at the Great Western Hotel, Paddington; Dr. Hitchman in the chair.

The following officers were present:

Dr. Hitchman, President for the past year.
Dr. Forbes Winslow, President Elect.
Dr. Thurnam, Ex-president.
Wm. Ley, Esq., Treasurer.
Dr. Bucknill, Editor of the *Journal*.
Dr. Campbell, } Auditors.
Dr. Prichard, }
Dr. Lockhart Robertson, Honorary Secretary.

And the following members and visitors:

| | |
|---|---|
| F. D. Walsh, Esq. | Dr. Fayrer. |
| Dr. Sutherland. | Dr. Sankey. |
| Sir C. Hastings, M.D., D.C.L. | Dr. Donald Mackintosh. |
| Dr. Sherlock. | Dr. Lowry. |
| Dr. Kirkman. | J. Terry, Esq. |
| Dr. Chevallier. | Dr. Manley. |
| T. N. Brushfield, Esq. | Dr. G. Stilwell. |
| Dr. Monro. | Dr. Harrington Tuke. |
| J. Millar, Esq. | Dr. Davey. |
| Dr. Boisragon. | Dr. Stevens. |
| J. Cornwall, Esq. | Dr. Paul. |
| Dr. Burnett. | R. Gardiner Hill, Esq. |
| T. Allen, Esq. | J. G. Symes, Esq. |
| F. D. Tyerman, Esq. | Dr. Arlidge. |
| W. G. Marshall, Esq. | W. Hans Sloane Stanley, Esq. |
| A. Richards, Esq. | Dr. O'Connor. |
| Dr. Tate. | Dr. Wood. |
| Dr. Blandford. | H. Sankey, Esq. |
| Dr. F. K. Fox. | Dr. Willett, &c., &c. |

The minutes of the preceding meeting (1856) were taken as read and confirmed.

The Chairman (Dr. Hitchman), in resigning the office of President for the past year, made the following address:

"Gentlemen,

In resigning again to your trust the office of president, which I have had the honour to hold during the past year, I beg to state that no incident demanding a special report from me has transpired, except the proceedings of the Society in reference to our associate, Mr. Millar. That case is, I believe, as well understood by the members as by myself, and yet a recapitulation of the chief facts may not be irrelevant to the business of the meeting. In Sept., 1856, Mr. Millar published a pamphlet detailing the perpetration of an act of injustice by the Committee of the Bucks Asylum. So unjust was the conduct of the Committee, as related by Mr. Millar, that men were reluctant to believe that a body of English gentlemen could be capable of such proceedings. The Association shared in this doubt. The superintendents of asylums had had large experience of the high character of the English magistracy, and had found in them a chivalrous sense of honour, and a love of open, even-handed justice and manly candour; and an especial abhorrence of mean, clandestine, and anonymous charges. They hoped, therefore, that the Committee of the Bucks Asylum would be no exception to this rule, and that the Committee would accord to Mr. Millar the right of meeting his accusers, of knowing the charges, and hearing the evidence which was brought against him. For my own part, I strongly believed this, because I knew the chairman of the Bucks Committee to be a gentleman thoroughly conversant with business, long and intimately accustomed to the management of public societies of a scientific and benevolent nature. I had heard his character portrayed in high terms, and, moreover, knew him to be associated by relationship and intimacy with a family for whom I have great reverence. I felt that all the facts were not before the public, and prior to calling upon the Association to protest against the proceedings of the Committee, I addressed the chairman. Subsequent events proved that I had erred in my opinion, and that sufficient allowance had not been made by me for the contagiousness of passion, and the fanaticism of corporate bodies. The following fact would be incredible, were not Mr. Millar in possession of the minute to vouch for its accuracy:

"19th Sept., 1856. *Extracts.*—At a meeting of the Committee, present, Thos. R. Barker, Esq., chairman; John Lee, Esq., LL.D., Christopher Tower, Esq., W. Lowndes, Esq., T. T. Bernard, Esq., C. T. Gaskell, Esq., J. T. Senior, Esq., W. Pennington, Esq., the Rev. A. P. Cust, the Rev. C. E. Gray, the Rev. R. Townsend,—*Resolved*, 'That it is the opinion of this Committee that the application made by Mr. Millar, for an extract of that part of Mr. Carrington's letter, on resigning his office of chairman of the Committee of Visitors of the Lunatic Asylum, dated 8th January, 1856, and entered on the minutes of the Committee, on the 15th January, 1856, be not acceded to.'"

And the following is a copy of another extract in Mr. Millar's possession:

"At a meeting of the Committee of Visitors, held on the 24th Oct., present, Thos. Raymond Barker, Esq., J. Lee, Esq., LL.D., William Lowndes, Esq., C. Tower, Esq., C. T. Gaskell, Esq., J. T. Senior, Esq., T. T. Bernard, Esq., the Rev. C. E. Gray, the Rev. A. P. Cust—'Mr. Millar applied for a copy of the charges made against him, which led to the resolution passed by the Committee on the 29th August last. The Committee decline to furnish the same.'"

On the reception of the letter from the Bucks Committee, disdaining to answer the simple inquiry which was made to it, the Committee of this Association felt that the time for a public remonstrance had come, and the protest which nearly all the members have subscribed was the result. As I adopted, rather than composed that remonstrance, I may be permitted to state that it was drawn up with much clearness and force, and received the approbation of nearly every member of the Association. Two or three gentlemen, from motives which I am not at liberty to explain, hesitated to attach their names to it, but from every superintendent (with one exception only) I received a courteous reply to my application for his signature.

Perhaps no document ever elicited so much unanimity of opinion. It was posted to every magistrate in the county of Bucks, previous to their meeting at Epiphany Sessions. Mr. Millar has stated, that it was only on the day of the Session, namely, Jan. 5th, that he was supplied with a copy of the charges preferred against him. To these charges Mr. Millar has since published a "refutation," which has been read probably by all the members of the Association. These must have rejoiced to find, that, notwithstanding all the aspersions which have been made upon Mr. Millar by a Committee of the Bucks Asylum, imperfectly acquainted with the proper management of a lunatic asylum, that Her Majesty's Commissioners in Lunacy, skilled and experienced in such matters, have since given public testimony to the value of his services, and to the creditable state of the institution. And while the Commissioners have done this, his professional brethren have, upon public grounds, and upon public grounds alone, come forward to protest against the injustice with which he has been treated, and to bestow upon him their sympathy and their aid.

For myself, I have seen Mr. Millar but once, and then only for a few minutes; yet I rejoice at the manner in which he has passed through this persecution, and beg to congratulate him upon the high position which he now holds in the opinion of his professional brethren, and in the estimation of the Commissioners in Lunacy, and of this Association. Few men have had the good fortune to be thus supported, when calumny and injustice have fallen upon them. Sustained by the inner consciousness of having acted well, such praise and such sympathy are a deep consolation and a rich reward; a guerdon of honour to himself, and a brand of perpetual shame to those who have wronged him."

Dr. Hitchman then resigned the chair to Dr. Forbes Winslow, the President Elect, who, in taking the chair, delivered an address, which we publish in another part of this *Journal.*

### TREASURER'S REPORT.

Mr. Ley proceeded to read the Treasurer's Report.

*Receipts and Expenditure for the Year ending July* 1, 1857.

| Receipts. | £ | s. | d. | Expenditure. | £ | s. | d. |
|---|---|---|---|---|---|---|---|
| By Balance in the hands of the Treasurer . . . | 27 | 18 | 4 | By Annual Meeting, 1856 . | 1 | 5 | 0 |
| By Annual Subscriptions paid to Treasurer . . . | 95 | 11 | 0 | By Printer's bills for Journal Numbers, 18, 19, 20, 21 . | 108 | 4 | 11 |
| By Annual Subscriptions paid to General Secretary . . | 10 | 10 | 0 | By Official Notices, &c. Postage | 1 | 7 | 4 |
| By Annual Subscriptions paid to Irish Secretary . . | 4 | 4 | 0 | By Expenses of President's Remonstrance in the Case of the Bucks Asylum . . | 9 | 11 | 8 |
| Subscriptions to Journal . | 3 | 4 | 6 | By Expenses of Irish Secretary | 0 | 4 | 0 |
| | | | | ,, ,, General Secretary | 4 | 19 | 6 |
| | | | | ,, ,, Treasurer . . | 0 | 19 | 6 |
| | | | | By Balance in hand of General Secretary . . . | 5 | 10 | 6 |
| | | | | By Balance in hand of Treasurer | 9 | 5 | 5 |
| Total . | £141 | 7 | 10 | Total . | £141 | 7 | 10 |

Correct. D. C. Campbell, Thomas Prichard, } Auditors.

The expenses had been very similar to those of the previous year, and the receipts were much the same. The report of the treasurer's accounts and expenses had been audited by the auditors, who would pronounce an opinion as to its correctness. The only point to which he had to call attention, as being something unusual, was a matter which had taken place at the last meeting. A committee was then appointed to act in cases of emergency (not formed from the whole body), but when it might be for the interest of members generally, they had power to call them together. That committee had acted in the case of Mr. Millar through the President, and some slight expenses were incurred, which received the President's signature, and the auditors thought it right to pass the account, which was made out separately, though it was but small, because the auditors, collectively, felt they could not say that the accounts were made up in the same way as in the former year. There was a balance in hand of 14*l*. 15*s*., and about 60*l*. outstanding debts. Many of the members had come forward to double their subscriptions, some honorary members had sent in two guineas instead of one, not knowing whether they were to pay for a future year; and in all cases there was an expression of good will that left no question as to the disposition to keep up the subscription. With reference to the balance which was due to the treasurer, a great part of it had not been asked for. He could, with propriety, repeat the expression which he had made use of at the last meeting, as to the perfect success of the society.

Dr. Campbell begged to observe that as to the accounts, he thought it would be found that a great number of subscribers were in arrears; some had not paid up their suhscriptions for two years, some not even for three years. He recollected that two years ago a motion was made, that unless the subscriptions were sent in and paid for the two years, notice should be sent to the parties so in arrear, and if, after notice so sent, the subscriptions were not paid, the defaulters' names should be struck off the roll of members. He thought the names of persons in arrear should be struck out after three years.

The President asked whether Dr. Campbell had any intention of making a motion to that effect.

Dr. Campbell said he would move, "That those who should not have paid up their subscriptions for two or three years, after notice being sent (if necessary), in two or three months, should have their names struck out."

Dr. Hitchman: There was a rule in respect to such cases.

Dr. Robertson said it was provided under Rule V.,

"That any member in arrear of his subscription more than twelve months after the expiration of the year for which it becomes due, and more than three months after application by the Secretary for the same, shall cease to be considered a member of the Association, provided no reason satisfactory to the annual meeting be assigned for the non-payment of such arrears."

Dr. Campbell would then simply move, "That the fifth rule of the Association be enforced;" and the motion being seconded, was carried unanimously.

Dr. Burnett begged to move, "That the treasurer's report be received."

Dr. Sherlock seconded the resolution, which was carried unanimously.

Dr. Sutherland begged to propose a vote of thanks to their late President. That gentleman had first raised the office of President to the dignity which it had now acquired; and he had by his admirable conduct in the chair, raised this institution very much in the eyes of the public. He thought the committee which had been established last year was of much use practically, and that the Association had much need of it in this way, that it was a check upon those who, as Dr. Winslow had said (and there were those persons), did not understand them. Unless they went together, they might have to encounter, not only cases like that of Mr. Millar last year, but others; and they might be liable to be crushed as an association formed for the most beneficent of purposes.

Sir Charles Hastings said it was with the greatest pleasure that he rose to second the vote of thanks to Dr. Hitchman.

The President having put the question, it was carried by acclamation.

Dr. Hitchman begged to tender his best thanks for the very kind manner in which the members of the Society had received the last resolution. He was indeed happy to find that his services had been deemed acceptable to the Society, and had elicited their so cordial approbation. As far as his efforts enabled him, it would be the dearest joy of his heart to see the Association prosper; and he hoped that feeling might be carried out which had been so eloquently referred to by their President.

ELECTION OF OFFICERS.

The President said the next business was the election of officers. They must first elect a *President* for the next year, and it would be open to any member to propose a gentleman for that office.

Dr. Bucknill rose and said: It fell to him to propose the name of a gentleman for President next year, who, he was sure, would reflect great honour and dignity on this Association. Important as this Institution had now become, still the presidency of a gentleman who stood so high, not only in this country, but throughout the world, in connexion with the treatment of insanity, he felt assured they would, in common with himself, regard as an event which would reflect honour upon and promote the interests of their Association. He meant Dr. Conolly. He believed it had been suggested that the Association should meet next year at Edinburgh. He mentioned this, it was true, incidentally, and perhaps it was not the right time to mention it; but the Association would probably meet at that great seat of learning and science, the capital of the north; and it seemed to him very important that they should there have at their head a man of Dr. Conolly's eminence, and that they should go with a staff of officers as strong as possible, in order to make the best of that occasion. He felt it quite unnecessary to eulogize Dr. Conolly, his name was so well known to all the gentlemen present, that he should content himself by proposing that Dr. Conolly be their President for the year ensuing.

Dr. Hitchman had great pleasure in seconding the proposition that Dr. Conolly should be the President for the ensuing year. The position of Dr. Conolly, his European fame, and the greatness of his character, eminently fitted him to promote the interests and dignity of this Institution. While some names needed elaborate eulogy, Dr. Conolly's name had ever been a household word with them all. By his earlier pursuits, he had fitted himself for the great task of enlightening the ignorant, soothing the sorrowful, and promoting the cause of truth. Let them look to his brilliant career at Hanwell. The Hanwell reports marked an epoch, they unfolded great facts in language of which the literature of the country might be proud. His able work on asylums and their management, he thought, stood as a monument of his fame. He therefore had great pleasure in seconding the resolution.

The proposal was carried by acclamation.

The President: The general way of proceeding was, after electing a President for the next year, to select *the place of meeting.* Therefore it was desirable to decide now where they should meet next year.

In answer to a question, Dr. Lockhart Robertson read Rule XII.

"Place of Meeting.—That the annual meeting be held either in London, or, if so agreed at the preceding meeting, or after circular to each member, in some provincial town or city where, or in the neighbourhood of which, there is a public asylum, or where some other object is likely to attract the members."

The President desired to know whether it was the pleasure of the meeting that the place of meeting should be Edinburgh, if the British Medical Association went there.

Dr. Stevens said he really did not like to hear their place of meeting spoken of in connexion with the possible movements of any other institution. He knew it was very inconvenient for many members of the profession to go to Edinburgh. He would therefore propose as an amendment "That they do meet in London."

Dr. Bucknill said that this was a matter which he thought ought to be decided by vote, being one in which the convenience of the majority of the members should have the greatest weight. He would therefore suggest that it would be best to vote as between London and Edinburgh, with the proviso that the British Medical Association met there.

Dr. Lockhart Robertson then moved, "That the annual meeting for the year 1858 be held in Edinburgh."

The President observed, that it was clear that Dr. Conolly had been elected President for next year; and the next question was, Where should they meet? He thought that Dr. Conolly would think it a great compliment that they should go, under his presidency, to Edinburgh to meet the British Medical Association; not that they should go in their tail, but *pari passu* with them. He thought they would materially reflect importance and dignity on each other. He would further recall attention to the fact, that they had among their body many very eminent gentlemen in Scotland, and he thought they should turn their steps occasionally in that direction. It was a pity to confine themselves to England. If those who were members of the British Association, as well as of this, went to Edinburgh, it would be a good opportunity for this Association. But the question was in the hands of the meeting.

Dr. Bucknill said he should second the motion of Dr. Lockhart Robertson, that they should meet in the city of Edinburgh next year.

The President said he had now to put the motion which had been made by Dr. Robertson, and which had been seconded by Dr. Bucknill, "That the annual meeting for the year 1858 be held in Edinburgh." On the question being put, there appeared for the motion, 17, against it, 3.

The President said he thought they might now congratulate themselves on having passed that resolution. The meeting would now proceed to *the election of a Treasurer*.

Dr. Robertson said he had the honour to propose Mr. Ley as Treasurer of the Association. No one of the officers of the Association had so often as himself come into contact with that gentleman, from the very nature of their respective offices. No man could devote more time and care to the funds and the interests of the Society than Mr. Ley. He had therefore great pleasure in proposing him as Treasurer for next year.

Dr. Boisragon seconded the motion, which was carried unanimously.

The President: They had now to proceed to another important business, and that was to elect *an Editor of their Journal.*

Sir Charles Hastings had great pleasure in proposing Dr. Bucknill as the Editor of their *Journal* for the ensuing year. That gentleman carried out the objects of this Association in the best manner, and he edited the *Journal* without any exclusive views.

Dr. Monro seconded the motion. He would only say that he could scarcely conceive a scientific *Journal* which could be more ably conducted.

The question being put, was carried with acclamation.

The President: They had now to elect Auditors.

Dr. Robertson said the Auditors were Dr. Campbell and Dr. Prichard; one of these gentlemen was re-eligible, but that the other must retire. Dr. Campbell had given extreme satisfaction, he understood Mr. Ley's mode of carrying out the accounts, and he hoped that Dr. Campbell would again be re-elected as one of the auditors.

Dr. Prichard declared he should much prefer Dr. Campbell being re-elected to himself.

Dr. Robertson: The rule was, undoubtedly, that one was re-eligible and that the other retired.

Dr. Prichard said he would at once retire, in order to secure the re-election of Dr. Campbell, who was so well acquainted with Mr. Ley's mode of keeping the accounts, and he begged to second the motion for his re-election.

Dr. Sherlock proposed Dr. Stevens, of St. Luke's, as the other auditor; and the motion having been seconded,

The President put the question, that Dr. Campbell and Dr. Stevens be the Auditors for the ensuing year.

Carried unanimously.

The next business on the paper of agenda was the election of a *General Secretary.*

Dr. Hitchman proposed Dr. Lockhart Robertson as General Secretary for the ensuing year.

Dr. Tuke felt great pleasure in seconding the motion.

The question being put, was carried unanimously.

The President said they had now to elect two other Secretaries —one for Ireland, and another for Scotland.

Dr. Bucknill proposed that Dr. Stewart be re-elected the Secretary for Ireland. Dr. Stewart is a gentleman who has taken a most active interest in the welfare of this Association. That interest he still retained, and he much regretted that from an accidental circumstance he had not been enabled to be present on that day. The fact was, that he had not been informed of the day of meeting sufficiently soon. He believed his absence would be generally regretted; and he would have been with them, he was sure, if he could.

Dr. Robertson with much pleasure seconded the motion. By an oversight of the printer's, Dr. Stewart had not received the intimation of the day of meeting until it was too late for him to be present. He had received it only two days before. He was requested by Dr. Stewart to convey to the meeting his extreme regret that he had so been prevented from being present.

Mr. Ley proposed, and Dr. M'Intosh seconded, that Dr. Browne be re-elected Secretary for Scotland.

### NEW MEMBERS.

The President said they had now to proceed to the election of New Members, and perhaps he might be allowed to propose as an honorary member of the Association, one of the most distinguished psychologists of France, who had paid the Association the compliment of coming over from Paris to meet its members. He referred to Dr. Brierre de Boismont, whose name must be as familiar to them as a household word, a man of European fame, of great personal worth, and of high attainments. He (the President) thought they would be guilty of an act of discourtesy if they were to overlook the fact he had stated, and not elect this gentleman one of their honorary members. They had no rule as to distinguished foreigners, but if he were the first they elected, they could not have selected a better man. He begged to propose this eminent man's name first in the list of honorary members to be this day elected.

Dr. Sutherland said he had much pleasure in seconding the proposition of the President. Dr. Brierre de Boismont had told him that he was most anxious to be introduced to the Association, but he was sorry subsequently to hear that he would not be able to be present at this time.

The question was put by the President, and was carried unanimously.

Dr. Robertson proposed as an honorary member, Mr. Hans Sloane Stanley, the Chairman of the Board of Visiting Magistrates of the Hants County Lunatic Asylum, who wished to become one of the honorary members of this Association. He hoped that if ever a future chairman of the Bucks Asylum took as high a position, they would elect him also.

Dr. Hitchman begged most cordially to second the nomination of Mr. Stanley. He occupied a great position, and the fact of Mr. Stanley coming forward, reflected honour on the Association which received him as an honorary member.

Resolution put and carried unanimously.

Mr. Stanley begged to acknowledge the great honour which had been paid him by the members of the Association, in electing him into their valuable Society. During the time he had served as Chairman of the Committee of Visiting Magistrates of the Hants County Asylum, he had always felt a deep interest in the progress of this Society. He had subscribed to the *Journal;* and though he could not say he had read all the various papers with which it was filled, yet he had read enough to excite his deepest sympathy in the institution, and to make him wish to become a member. He trusted that the harmony which existed between the committee over which he presided and the medical superintendent was so well established, that no such circumstances as those which had been referred to by the late President would arise. Such was the cordial feeling between their Committee and Dr. Manley, though they had different duties to perform, they would respectively carry out those beneficial improvements which were suggested, from time to time, in the treatment of those unfortunate persons who were placed under their care. He had not come there, however, to make a speech, but as a listener, and a promoter of that science for the furtherance of which they were assembled together.

The President: They had now twenty-five ordinary members of the Association to propose; and what he would venture to suggest would be, that the Secretary should read over the names, with the names of the proposers and seconders, and that then, in order to save valuable time, they should elect them *en masse.*

Dr. Robertson then read the following list:—

ORDINARY MEMBERS.

1. Richard Adams, Esq., M.S. Cornwall County Asylum, Bodmin.
2. J. Bartlett, Esq., Sussex House, Hammersmith.
3. J. J. Blake, Esq., M.B., Essex County Asylum, Brentwood.
4. Dr. Blandford, 7, Grove, Brompton.
5. Dr. Dillon, V.P. Ballinasloe District Asylum, Ireland.
6. Dr. Duncan, Farnham House, Finglas, Ireland.
7. Dr. Francis Fox, } Brislington House.
8. Dr. Charles Fox, }
9. F. Gould, Esq., County Asylum, Hants.
10. J. Hawkes, Esq., Wilts County Asylum, Devizes.
11. Dr. C. Howden, the Royal Lunatic Asylum, Edinburgh.
12. J. Humphrey, Esq., M.S. Bucks County Asylum, Aylesbury.
13. W. Langley, Esq., Rivertop House Asylum, Uxbridge.
14. Dr. D. M. M'Cullough, the Royal Lunatic Asylum, Edinburgh.
15. Dr. Peppard, Bushy Park, Limerick.
16. J. Philipps, Esq., Bethnal Green, London.
17. Dr. Rogan, M.S. to the Londonderry District Asylum.
18. Dr. Andrew Ross, Portsmouth.
19. Dr. Stilwell, Moorcroft House, Uxbridge.
20. J. P. Symes, Esq., Devon Branch Asylum, Exmouth.
21. Dr. Tanner, Charlotte-street, Bedford-square.
22. Dr. Tate, St. Luke's Hospital, London.
23. R. Walker, Esq., County Asylum, Chester.
24. F. Wilton, Esq., County Asylum, Gloucester.
25. Dr. Andrew Wynter, Brompton.

The President put the question, whether it was the pleasure of the meeting that the gentlemen whose names they had heard read should be elected ordinary members?

Dr. Davey rose to propose Dr. O'Connor. He was not engaged in their particular department of the profession, but he was much interested in the treatment of the insane, and he wished to become a member.

Dr. Robertson said he had to state that when this gentleman's name was brought before the Committee, an objection was taken to him, from his not being specially engaged in this department of medicine. The rule, as relating to members, was, "That the Association do consist of medical officers of hospitals and asylums for the insane, public and private, and of legally qualified medical practitioners, otherwise engaged in the treatment of insanity."

The President: In whatever might be done, he begged to say, on behalf of the Association, that there was no personal feeling towards Dr. O'Connor. The rule, as read by the secretary, existed, and it was a stringent one, and one which they were bound to adhere to. He was sure that Dr. O'Connor would see that, in adhering to the prescribed rule, nothing personally offensive was intended to him.

Dr. Stephens would beg to second the name of Dr. O'Connor.

The President: It is necessary to go to the ballot. Dr. O'Connor would clearly understand what was the motive of the Committee in opp6sing his election. There could be no personal feeling with regard to himself, but if they had a law, he felt they should adhere to it stringently; a principle which, he was sure, Dr. O'Connor would appreciate.

Dr. Thurnam suggested whether a resolution could be put as to adhering to the recommendation of the Committee as to the list of the new members to be elected?

The President: Would any one move an amendment to this effect?

Dr. Robertson then moved as an amendment, "That the recommendation of the Committee, as related to the list of new members, be adhered to."

Dr. Thurnam seconded the amendment.

The question was then put, and on a show of hands being taken, there appeared—

| | |
|---|---|
| For the amendment . . . . . . | 18 |
| Against it . . . . . . . . . | 2 |
| Majority in favour of the amendment . | 16 |

The original motion was therefore lost.

ALTERATION OF RULES.

Dr. Tuke said he rose, in pursuance of the notice which he had given last year, to move, "That the names of proposed honorary members be printed, and sent round with the circular convening the meeting of the Association." The inconvenience of the present practice was, that they were not supplied beforehand with the names of the new members to be proposed. He thought it was a bad compliment which they paid to their honorary members to be in ignorance of whom they might be; and, on the other hand, there was no opportunity to object to persons at the moment they were announced.

Dr. Campbell seconded the resolution, which was carried unanimously.

### ACTING SUB-COMMITTEE.

Mr. Ley said he rose to move the re-appointment of the acting Sub-Committee. The matters to which this Committee applied itself were peculiar. Circumstances might arise which would make it desirable that this Committee should act, and summon the general body, when it would be inconvenient to any private member to take that duty upon himself. Thus, they were likely to have questions sent to them during the session of Parliament, when the Society itself would be incapable of acting. In the course of last year they made the President take upon himself the correspondence with the Committee. A small number of persons thus acted together, and agreed on the mode of proceeding. By this means much more work was done than by waiting to have the concurrence of some 120 or 130 persons who were members of the general body. His proposition was, "That the acting Committee of last year be re-appointed."

Dr. Sherlock begged leave to second the motion, knowing, as he did, the admirable manner in which the business of the Sub-Committee was carried on. He sincerely concurred in all that had fallen from Mr. Ley.

The motion was then put. Carried unanimously.

Dr. Tuke having been called upon by the President, proceeded to read the following paper:—

### OBSERVATIONS ON THE TREATMENT OF INSANITY, WHEN REFUSAL OF FOOD IS A PROMINENT SYMPTOM.

Mr. President and Gentlemen,—It is not without some diffidence that I venture to bring before this meeting, numbering, as it does, so many of the most distinguished practitioners in our department of medical science, views of my own, on a subject that must be so familiar to them: or that I attempt any description of a symptom of insanity that is so often seen, and which all of us are constantly called upon to meet. But I have found in private practice so great a diversity of opinion amongst medical men as to the treatment of this particular symptom, refusal of food—our text-books on the care and cure of deranged minds pass over the subject as one of so little importance; I have found it so impossible to obtain any information on the point, except scattered through the medical reports of asylums, which our Association may well be proud of, as containing all that is most valuable in the practical treatment of insanity—

that I believe I shall be doing a real service to medicine in bringing the question before this assembly. My own practice is comparatively of little importance: my object is rather to elicit and place on record the opinions of gentlemen so well qualified to pronounce judgment; whose dissent from my views would incite me, and perhaps others, to still further investigation, and whose concurrence in my conclusions would set the matter at rest.

I do not propose in these remarks to bring forward any new theory, or strange method of treatment. My object is to attempt a classification of those cases where refusal of food is a prominent symptom, founded on the real or presumed causes of such refusal: to point out the treatment necessary for each division; to discriminate those in which forcible alimentation is or is not justifiable; and lastly, to point out the various methods that may be adopted for this purpose, and the reasons that have induced me to choose the particular mode of treatment I myself prefer.

I divide those cases in which repugnance to nourishment or inability to take it exists, into five divisions, more or less distinct from each other. Disinclination to food in the insane may arise from—

1. Simple dyspepsia.
2. Delusion as to food itself, or to their power of taking it.
3. Suicidal tendency, or wound of gullet after an attempt at suicide.
4. Stupidity, inertness, idiotcy.
5. Special organic lesion in the brain or other internal organ.

I shall consider each of these classes separately, and although these divisions may not embrace all the cases that may arise or have been met with, and one may not often occur uncomplicated with some other, I believe such a classification will be found practically useful, and will, by clearly defining the nature of the case I am speaking of, enable me to defend myself against the charge of erring on the side of those who advocate mechanical interference in all cases where food is refused, or with those who think the forcible administration of food usually unnecessary, or even cruel.

I need not dwell on the first of my divisions; the symptoms are those familiar to the general physician. It is, however, most important that we should recognise the symptom, having the treatment of patients who are so often unable to explain their wants, or justly describe their sensations. Dyspepsia, superadded to chronic mental disorder, will frequently change for a time the character of the disease, induce new delusions, or add strength to old ones. The forcible administration of remedies in these cases may sometimes be necessary, but of food scarcely ever; and it is terrible to think that we may ignorantly inject into the stomach of an invalid suffering from headache, mania, or gastrodynia, an indigestible meal that will probably add to his sufferings, or even induce severe constitutional disturbance.

I shall not now enter on the treatment of dyspepsia superadded to chronic insanity, a subject I hope to have the honour of bringing before you on a future occasion. It is of frequent occurrence in private practice. It may sometimes induce repugnance to food, endanger life from exhaustion, and require mechanical feeding, but I have never seen such a case; and those practitioners make a grand mistake who sedulously pour gruel or beef-tea down the throats of those unwilling to eat, without investigating the causes of their reluctance. Much mischief may thus be done, and I believe to this indiscriminate use of the stomach-pump the objections that some of our first physicians have to its employment is mainly attributable.

The second group of cases, those in which food is refused under the influence of specific delusion, is the most ordinarily met with, and, fortunately, the most amenable to treatment. Such delusions are not often persistent, and the repugnance to food may usually be overcome by gentle and patient persuasion. Stratagem will often succeed, where you have any clue to the nature of the delusion. Medical treatment will frequently overcome such fancies, and of course obviate the necessity for further interference.

The delusions giving rise to refusal of food are sometimes most ridiculous—more often painfully distressing. The idea of poison being administered is, perhaps, the most common. Patients under my care have frequently refused food for this reason; and would thus, as it were, starve themselves to save their lives. Metallic taste, especially that of copper, is not unusual. There is, probably, always dyspepsia present in those cases, and the deranged secretions should be appropriately prescribed for. I have seen great mischief arise from drugging or tampering with the food of insane patients, a practice too frequently resorted to. A patient so treated will lose all confidence in those around him. In some cases it gives rise to an entire refusal of sustenance; in many it is the origin of these illusions of taste. Of course, I do not mean that such treatment is not sometimes useful. The stomach or the intestines may be the seat of disorder giving rise to this form of delusion. A patient now under my care believes that his stomach is turned inside out. Sometimes this idea will prevent his eating for twenty-four hours; but such abstinence relieves the uneasy sensations, and the use of bismuth and vegetable tonic infusions prevents the symptom becoming more severe. The delusion itself has existed for ten years.

The idea that voices are heard warning them against food is a frequent and dangerous symptom in deranged patients, often ushering in or attending suicidal mania. In all these cases active medical treatment is most essential. A lady very recently under my care was, at the commencement of her attack, obstinately bent on suicide, requiring the constant presence of an attendant. She was fed with a spoon for several days, but with great trouble and difficulty. She would give no reason for her abstinence. A great amount of nervous tremor, want of sleep, fits of weeping, marked one of those cases, which Dr. Hitchman, of Derby, our last President, has pointed out to us as being so especially benefited by opium. After taking it in the form of Battley's solution for a few days, the repugnance to food ceased. Under the persevering use of this remedy, her melancholy disappeared. She is now rapidly recovering, and tells me that her objection to food arose from imaginary voices thundering in her ears warnings against her taking it, and telling her it was "bathed in human blood."

I need not, before my present hearers, enlarge upon the various recorded plans by which patients have been seduced, or surprised into taking food. Dr. Conolly, in his "Clinical Lectures at Hanwell," used frequently to mention the case of a man who had persisted in refusing food for a dangerous length of time, but at length eat heartily of a mighty seed cake, which the steward, with the view of tempting him, caused to be cut up and distributed in his presence, without any apparent wish that the patient also should share it. This is a useful hint to the practitioner; in such cases too much anxiety defeats our object. An affected indifference will often disarm the suspicions of a patient, and induce him to give up his intended abstinence.

Esquirol pretended to flog a patient of his who obstinately refused food, telling him that if he persisted in acting like a naughty child, he must be treated like one. The expedient for the time succeeded. The Bourbon prince who imagined himself dead, and was induced at last to eat by an invitation to meet some distinguished pretended ghosts, who assured him, by precept and example, that eating was quite compatible with his and their position, is familiar to us all. I question the wisdom of such a plan; and I believe it is recorded, that the poor prince, undeceived as to his companions, at last died a victim to his delusion, and to the prestige of his rank, that interdicted the employment of forcible means of nourishment.

A change of diet, or allowing the patient to choose his own food will sometimes be beneficial. A young Spanish gentleman under my care would not eat. In the hope of ascertaining the reason of this resolution, I invited him to dine with me. On his plate being handed to him, he rose from the table, pale, trembling, and with all the marks of the most unfeigned abhorrence. "*Mon Dieu!*" said he, "it is a woman's flesh you give me." I had now a clue to his delusion. My suggestion that eggs were not open to this objection was well received. His repugnance to other food soon wore off, and under appropriate medical treatment he rapidly recovered.

Another patient, a boy of eighteen, whose refusal to take solid food began to give me great uneasiness, I induced to eat by inviting him to help me dress some mutton chops, which I affected to take with great mystery from my own larder, in the absence of the cook. He entered into the joke, and, without any pressing, eat more than his fair share; and as he had not tasted food for more than thirty-six hours, I was delighted to see him eat. Badly dressed chops were never, perhaps, so much enjoyed. From this time his recovery commenced, and he is now perfectly well. I mention these cases, because it is obvious that, these plans not succeeding, either of them would have been proper subjects for the forcible administration of food. In their weakened physical and mental condition, a few hours' longer abstinence might have been a serious obstacle to their ultimate recovery.

Sometimes persuasion, with a little gentle force, will induce a patient to take food, in spite of his delusion; and finding no ill result follow, the persistence in abstinence is overcome. But it is only to experienced and kind hands that this experiment can be safely entrusted. No servant should be allowed to threaten the stomach-pump, or to employ even the slightest force, without the presence or the express sanction of the physician in attendance. Ill-judged efforts at feeding increase the repugnance, which tact and gentleness might overcome. Still the more grievous error appears to me to be in delay. The valuable aid of the stomach or nasal-tube is neglected till exhaustion has set in, and even if life be preserved, the mental disorder has become more deeply rooted, and the patient remains a chronic case, to be daily fed, who under early medical treatment would have recovered his mental, as well as his corporeal strength.

Change of scene, and of the immediate attendant, is worthy of trial. I have seen a patient who refused food obstinately in his own sitting-room, dine with appetite in the company of others. Attention to the quality of the food, to the way it is cooked and presented to the patient, is in private practice absolutely essential. I should take care that the soup I was about to inject through the stomach-pump was well served up, as though about to be taken by myself. I have seen at the last moment a patient elect to eat, rather than be forcibly fed; and he is more likely to do this if the food offered is not a disagreeable mess of beef-tea and gruel, such as he would not have touched when in his usual health.

The third of my divisions—cases in which there is a determination to die by starvation—gives, perhaps, the most anxiety to the medical man. At any moment the desire for self-destruction may take some other form. The great point in the treatment of other cases is to decide when you have carried persuasion far enough, and the exact time at which you must resort to mechanical and forcible feeding. The age, the constitutional strength, the habits of life of the patient, must guide us here. It must be remembered, that if insanity is essentially a disorder of debility, in suicidal cases, as a general rule, there is more particularly an exhaustion of nervous power, and that each hour's delay diminishes the chance of the patient's recovery.

The length of time for which abstinence can be borne is sometimes extraordinary. In one remarkable case, a man existed for seventeen days without food. Captain Chesterton, in his "Revelations of Prison Life," gives two instances of voluntary abstinence from food for thirteen days, without injury. I do not myself wait in these cases till the pulse begins sensibly to flag; there is no harm in being too soon. The longest time I have ever ventured to delay has been four days. My usual rule is not to wait more than forty-eight hours.

Long abstinence in some constitutions produces a train of symptoms very apt to mislead the practitioner who has not watched the progress of the case. Excitement comes on, a state analogous to that seen in *delirium tremens*, strange visions pass before the patient, horrible sounds are heard; there is mania without inflammatory symptoms, prostration with excitement. The remedy for this state of things is, the forcible administration of food in small quantities, and even stimulants. The following case illustrates this form of disorder:—

In the summer of last year a lady, travelling abroad, lost her only daughter. Her grief took the form of religious melancholia. She was brought to London to consult Dr. Conolly. Soon after there were several attempts at self-destruction; then an entire refusal of all nourishment. Excitement now came on, with mania such as I have described. At this stage, Dr. Conolly recommended her removal to my house. No food had been taken for two days; for two days more everything but water was refused. Raving continued, but dangerous exhaustion was becoming evident. On the fifth day we determined on injection of food into the stomach. I sent through a tube introduced through the nostril a small quantity of beef-tea thickened with isinglass, and two ounces of sherry. Within six hours the raving ceased. For three days afterwards food and medicine were taken without much repugnance, but there were frequent attempts at suicide in other ways. Forced nourishment was only once more necessary. The tincture of Indian hemp and opium were freely used in the after treatment; and this lady recovered perfectly, and has remained since perfectly well. Writing to me from Wiesbaden lately, this lady, after many kind and grateful sentences, adds, "To you and to Dr. Conolly I owe my life."

It is singular how long patients will sometimes permit themselves to be forcibly fed, rather than take food voluntarily. I have fed such cases through a tube for many weeks, and cases are on record where it has been necessary to do so for years.

I may mention here, that it is important to vary the aliment introduced. Arrowroot, gruel with or without milk, beef-tea thickened with isinglass, or with flour, or with the yolk of eggs, are all available. To my friend Dr. Hodgkin I owed the suggestion, in one case where feeding was necessary, of pounding roasted chicken in a mortar, adding milk, and rubbing it down to a cream, which passed easily through the smallest tube. Thus imitating, as nearly as possible, the effect upon the food produced by mastication and insalivation. In the case of a patient at St. George's Hospital, whom it was necessary to feed daily for twelve months with the aid of the stomach-pump, a tube of double size was procured; and through this meat and vegetables were passed down the œsophagus, cut up in the ordinary way. The man did not appear to suffer under this treatment. I rather imagine that those cases which the opponents of forced alimentation adduce, of patients who have sunk with symptoms of atrophy and exhaustion in spite of the stomach-pump, have too frequently either been left too long uninterfered with, or have not had a judicious variety of diet. I do not believe that a patient of depressed vital power would live for any length of time upon beef-tea alone, and his sinking would be an argument, not against his being fed by force, but against delay in the first instance, and against the administration of improper and insufficient aliment.

There is one important point to remember in these cases of refusal of food; the intention of suicide will rarely be confessed. If, therefore, the cause of the refusal is not ascertained, you must consider such a patient dangerous to himself, and watch carefully against efforts at self-destruction repeated in some other shape.

As the result of such attempts, wounds of the throat come sometimes under our notice, but more frequently under the care of the hospital surgeon, as the result of suicidal attempts in *mania a potu*. Mechanical feeding will of course be required, and caution and careful manipulation are essential; a small tube should usually be employed.

The fourth class of cases is easily disposed of. They are not numerous, their diagnosis is easy, and their treatment obvious enough. In the case of idiotcy and imbecility, spoon-feeding will generally answer the purpose. Should it fail, the stomach-tube must be resorted to. Dr. Leon de Verga, usually opposing all attempts at forcibly feeding the insane, excepts this class of cases. "I do not call it, in this case," he says, "forced," but "artificial alimentation." As he admits they should be fed, I will not make any objection to his nomenclature.

Special lesion of the brain, or organic disease of internal organs, occasioning the refusal of food, I have made the last of my divisions. There can be no disorder that requires more careful study, or that places the medical man in a more painful position. On the one hand, interference may add to the agonies of the certainly dying patient; on the other, how distressing to witness prolonged suffering without an attempt to relieve it.

Instances of disease must too often come before us, in which we are forced to confess how unavailing are all the resources of our art; but it is a heavy responsibility to doom, by non-interference, a patient to a certain, a painful, and a lingering death, without an effort to save him. And I am by no means certain, that in some recorded cases that have been left to die, the organic changes adduced to support such practice may not have been caused by long starvation. The effect has been mistaken for the cause. The motives of many of those who think the forcible administration of food an extreme measure are worthy of all respect. They shrink from anything like violent or severe treatment, as cruel and unjustifiable. At the same time, I must think them mistaken in their views. Dr. Leon de Verga writes an essay against the practice of feeding a patient contrary to his will. Would Dr. Leon hesitate to recommend tracheotomy, as a last resource, in a child dying with croup? I think not. And yet the same objections apply, and in a stronger degree. The little sufferer cannot consent, the pain is great, the operation is usually unavailing. In my own practice, if I considered that ulcer of the stomach, or intus-susception of the intestines, rendered alimentation unavailing, I should call in the general physician, or the operating surgeon, and even then urge the propriety of forced alimentation, as affording the last and only chance.

In the case of an old gentleman of weak physical power, who had been long insane, and whom I was attending with Dr.

Hodgkin, we suspected internal cancer. The patient could tell us nothing; he had all the appearance so characteristic of scirrhous disease. He took fluids freely, but obstinately refused all animal or solid food. He had become emaciated to a frightful degree; and, as a last resource, I injected some egg and wine into his stomach, with but little hope of any beneficial result. However, he seemed to rally, and in eight hours I repeated the operation. In the course of a few days he was walking about, comparatively strong. This feeding was at intervals necessary for about a month. He then began to take nourishment as usual, gained flesh, and seemed out of danger. At the end of seven months the same symptoms again appeared. All our remedies failed to do any good, and he died in a state of the greatest emaciation I have ever seen. An examination of the body after death showed us the stomach, reduced to one half its natural size, a thickened band embracing it, forming the appearance known as "hour-glass contraction;" the mucous membrane throughout was pale; the other organs of the body were apparently sound. There was little information gained by the examination of the brain. This gentleman's life was at least prolonged by our treatment; and the only thing to be regretted was, that we did not resort to the forcible administration of food earlier in the first attack.

I had the misfortune to have one very painful case under my care, which I bring before you—first, because it is an example of what I mean by repugnance of food arising from special disease of brain; and secondly, because it is a form of disorder which has been recently exceedingly well described by Dr. Bell; so graphically, indeed, as to have become known in America as "Bell's disease," but which I have never seen noticed before, except in Mr. Ley's Report of the Oxford Asylum for 1854, where he describes something like it as occurring after *delirium tremens*. A young country gentleman, of strong physical power, was brought to my house, suffering under a paroxysm of acute mania. He refused all solid food, though he took some little nourishment in the shape of barley-water, tea thickened with isinglass, and such things as occurred to us at the time. Several of the first London physicians and surgeons saw him with me. Forced alimentation was thought of, but we were agreed as to its being unadvisable, and in ten days my patient sank exhausted. The lungs had been resonant throughout, but breathing had seemed confined to their immediate apices; the respiratory sounds were scarcely audible; there had been intense inflammatory symptoms about the head, but these appeared to yield to treatment, and the immediate cause of death was considered to be pneumonia. On opening the thorax the lungs seemed too large for their bony cavity; the air-cells were distended, and, although healthy as to structure, were infiltrated in parts with frothy serum. The other viscera were perfectly healthy; the brain was not examined. These conjoined symptoms, functional disease of lung, and repugnance to food, appear to me to point out clearly the nature of the attack—acute inflammation of the membranes at the base of the brain, *involving the origin of the pneumogastric nerve.* The same in a chronic form might explain the want of inclination to food, associated with slow respiration, in some cases of melancholia; but I rather throw out this for the investigation of our Association. I do not wish to start a theory not immediately connected with my subject. The suggestion is, at all events, worthy of consideration; and, as far as I know, the coincidence of the symptoms have not been in any way explained, or even specially noticed. If I saw such a case again, I should recommend counter-irritation to the nape of the neck, and treat it generally as one of inflammation to the base of the brain, without reference to the lung-symptoms.

From the foregoing remarks, it will be easily seen that I hold decided opinions as to the propriety of forced alimentation in most cases of refusal of food, and that I strongly advocate the early adoption of this mode of treatment, before the strength fails, and fatal exhaustion is imminent. I quite agree with M. Emile Blanche, who says, in a letter published in the *Union Médicale,* in answer to some one who had decried the importance of mechanical interference, or in some case had neglected to give it a trial: "*Ce n'est pas sans un douloureux étonnement que l'on apprendra que les médecins en sont encore reduits à rester spectateurs désolés, mais impuissants, de l'agonie des malades.*" It is, indeed, with a sad astonishment I hear forced alimentation objected to by many eminent men; and I believe it is partly because its advocates have not clearly defined the cases where it is essential, have not dwelt sufficiently upon the importance of its early adoption, and have not taken pains to simplify their instruments, and to render the operation of feeding as little as possible distressing to the patient.

Of the various modes of forced alimentation, and of the forms of instrument used for the purpose, I have not now to speak.*

* At the conclusion of this division of his excellent Paper, Dr. Tuke showed to the Association a collection of instruments, and explained the different modes of treatment in use at home and abroad. The members of the Association expressed themselves highly gratified with the Paper, and requested that it might be published *in extenso* in their Journal. This, of course, is a rule with all papers thus submitted to their Association. In the early years of the Association, papers like the above were read at its meetings, to which they imparted a scientific character; but since the resuscitation of the Association, which has taken place during the last four years, no such papers have been read, until Dr. Tuke has this year revived

Dr. Davey said he was sure they were all much indebted to Dr. Tuke for his very interesting paper, but he wished to make two or three remarks upon the subject of compulsory alimentation. Cases had occurred within his own experience where that principle had been successfully carried out. One case he would mention in which such was the determination to resist food, that the patient must have died if she had not been sustained by nourishment supplied through the rectum, but she was restored to health by this treatment. Some five years ago, when he became the proprietor of an asylum, a lady, who for ten years had been kept alive by mechanical appliances, and who died about two years ago, had been fourteen years, although under the influence of powerful delusion, sustained by this means. She was kept alive by the introduction of food into the stomach, not with a tube, but with a funnel which was outside a pipe; the funnel was about the size of two hands, and she was thus kept alive for fourteen years. That was a fact which he considered to be worthy of record. There was another kind of case which bore relation to those mentioned by Dr. Tuke, which came under the head of hysteria, a disorder in girls which sometimes prompts them to refuse food, but to go away and consume food in some corner in secret. In the case he referred to, he accepted as truth what the girl told him as truth; but it came to his knowledge that she did eat on a certain occasion. He acted still upon the patient's assertion as truth, and it was only necessary to apply the remedy once. He said to her, "Will you refuse food? Then you must be fed artificially." She was fed accordingly, and the poor creature never gave him any more trouble. He considered she was cured by the inconvenience to which she was put. It set up a new action in her volition, she took her food quietly, and recovered.

Dr. Wood said, that as a London practitioner he had had a large number of patients who refused their food; and he had been rather startled at hearing such an authority as Dr. Conolly say that it was a rare thing for insane patients to require the application of stomach-pumps. Many curious cases of this character had come before him. It was necessary the first time to observe closely the bodily powers of the patient; and in the second place, to judge whether there were not peculiar features in the case which should lead the medical man to consider whether he ought not to hesitate to introduce food artificially. At the same the practice by favouring the Association with his excellent and practical essay. The discussion elicited was also in the highest degree valuable and interesting, although it by no means exhausted the subject. Dr. Tuke had only time to read an abstract of the concluding part of his Paper. He has, however, kindly promised to supply us with the whole of the remainder, so that it may be published in our next number.—Ed. *Asylum Journal.*

time, it was most important to introduce food at an early period; it should be determined on as soon as possible. In the criminal department of Bedlam, M'Naughten laboured under this delusion. He took it into his head that he would take no food. He was at the time in good health; there was no reason for the delusion on that score. He did not appear to be under any other than his ordinary delusion about the Tories having ill-used him, but he resolutely refused his food. He watched him for some days, and at last he became thinner, and eventually he was compelled to use the stomach-pump. He then said to the patient, "You must not die under my hands." He nevertheless still refused his food, and he was fed for about a fortnight, and gained flesh. At last it became almost a matter of joke between him and M'Naughten, who saw his folly, and eventually took his food without any trouble. There was another man in the same department, who, whenever he had to be fed, would be fed by the stomach-pump. He was determined to have his food in no other way, and actually introduced the tube into his mouth himself. There was no reason to oppose him, or he would have starved himself; and he soon gave up the notion when he found that he was not opposed. After all, the main point which they had to consider was the bodily powers of the patients, and the mode of introducing food must depend upon circumstances. There were sometimes circumstances of difficulty in the application of the stomach-pump; but with the nose-tube they could not introduce food sufficiently fast. Now, Dr. Tuke had referred to the pounding of meat, and yet the patients' commons might be reduced to pulp without much difficulty. He thought it desirable, also, that the food should not be limited to one kind, except where the patients were fed more than once a day. It was necessary to introduce farinaceous matter, for that they would get fat upon, and it was certainly more easy of digestion. There were patients who would die, let what might be done for them; but his feeling was that no medical man ought to let them die of starvation; they were bound to take every means, till they saw a man must die in spite of all their exertions. There was an impression abroad, that where patients were weak it was cruel to force anything in the shape of aliment; but he thought that, where a patient was dying, they were bound to administer food artificially, where it was necessary. There were many reasons, he contended, for the use of the stomach-pump, and but few for conveying aliment through the rectum.

The President asked Dr. Tuke whether, where a patient obstinately refused food, and struggled violently, and he were put slightly under the influence of chloroform, the patient then became facile to the introduction of the tube? as in some cases he believed, where patients struggled excessively, and were brought under the influence of chloroform, the tube was then introduced with comparative ease and success.

Dr. Tuke: There could be no doubt chloroform lessened resistance, and the tube would be more readily handled. Dr. Sutherland had tried this treatment with success. Dr. Davey had cited a case in which he had injected food into the rectum for many months, and the patient recovered. There appeared to him many objections to this practice. Dr. Davey had not stated the reason for this mode of treatment.

Dr. Davey: She was pregnant, and there was constant vomiting.

Dr. Tuke: Then the injection by the rectum was the last resource, and the case hardly bears on the question at issue. It is certain that life can only be supported for a limited period by this mode of nourishment. In cases of hysteria firmness was essential; but, in his opinion, threats should be avoided. Forced alimentation was a remedy, and not a punishment. He had to apologise for the length of his paper, and to thank the members of the Association for their kind attention.

Dr. Wood wished Dr. Tuke to understand that when he once told the patients they were to be fed by the stomach-pump he was not deterred by any consideration whatever in carrying out the threat.

The President had known cases where he had said to the patients that, unless they took their food rationally, the stomach-pump would be used; they have immediately taken their food, and continued to do so without the necessity of using it.

Dr. Tuke would here suggest, that he would not threaten the forcible administration of food unless he had the instrument open by him, to imply, "You mean resistance: I am prepared." He had never found any difficulty from resistance when using the nostril-tube.

Dr. Sherlock said he had known some cases in Edinburgh of delusion, acute excitement, and others, where it was necessary to have recourse to forced alimentation. In many cases where it was formerly used they gave chloroform, and the patients took it themselves; but in a proportion of the cases they only took the forced alimentation under the influence of chloroform.

The President: Dr. Tuke had made some observations upon medicating the food of the patients, a system advocated by Dr. Browne—namely, that of giving jalap in cakes, and senna in tea or coffee. And he also must say that he had seen very disastrous consequences resulting from this practice. The taste became nauseated, the patients soon discovered there was something noxious in their food, and something different from what they had expected to taste. They thought it extraordinary food, and hence they imagined that an attempt was being made to poison them. He thought that all practitioners should be very cautious how they meddled with such a system, which tended to create obstacles to the recovery of the patients.

Dr. Sankey: As to the administration of food artificially, it might be morally effected by having a large apparatus at hand, without using it; but the more simple the instrument the better. He had had a case where he had recourse to forcing the food. The patient continued in that case for three months with great resolution to refuse food. At last it was found that the application of two spoons was the most effective mode of proceeding—better than any amount of persuasion. One spoon was forced into the mouth to keep it open, and to keep down the tongue. The appearance of a large stomach pump, with its brass fittings, had an effect. The introduction of a tube into the nose might sometimes act well; but two spoons, he was of opinion, were often the best instruments.

Dr. Sherlock said in chronic cases it would suit very well.

Dr. Sankey: If there was a struggle, a gag must be placed in the mouth, and a simple spoon used. He should prefer a wooden one.

Dr. Wood: He presumed that it would take a long time to convey a sufficient quantity of food into the system by the mode now suggested.

Dr. Sankey replied that he would undertake to administer a pint of beef-tea in a quarter of an hour or less.

On the motion of Dr. Bucknill, seconded by Dr. Campbell, a vote of thanks to Dr. Tuke for his excellent paper was carried by acclamation.

The President informed the members that Messrs. Tyerman and Marshall, the Superintendents of the Middlesex Asylum at Colney Hatch, had a communication to make to the Association.

Mr. Tyerman then said that the Committee of Visitors of the Asylum at Colney Hatch had requested him and his colleague to communicate to the Association an invitation to visit and inspect that asylum on the morrow. A convenient train would start from King's Cross at 12.25; and after their inspection luncheon would be prepared for the members in the board-room.

The President said that the Association felt greatly obliged by this courteous invitation, and he did not doubt that many of the members would avail themselves of it.

Dr. Wood observed, that they had had a large and long meeting, and much trouble had been entailed on their respected President, to whom, on behalf of the meeting, he begged to tender their best thanks.

The proposition was carried by acclamation.

The President replied: He accepted the compliment which had been paid him. It was with satisfaction to himself, pride, and pleasure, that he had had to preside over so large and influential a body of gentlemen connected with the treatment of the insane.

Dr. Robertson, on the part of the Committee, gave notice that they would, at the next annual meeting, propose certain alterations in Rule II., and also in the designation of the Association.

The meeting then adjourned.

In the evening, the members dined together in the hotel, and were joined by Dr. Copland, and Mr. Gaskell, one of the Commissioners in Lunacy [an honorary member]. The arrangements of the hotel, the dinner, wines, &c., gave the utmost satisfaction.

THE PRESIDENT'S CONVERSAZIONE.

On the evening of the 1st of July, the President (Dr. Forbes Winslow) received the members of the Association at a *conversazione*, at his residence in Cavendish-square.

